# The Contribution of Artificial Intelligence in Achieving the Sustainable Development Goals (SDGs): What Can Eye Health Can Learn From Commercial Industry and Early Lessons From the Application of Machine Learning in Eye Health Programmes

**DOI:** 10.3389/fpubh.2021.752049

**Published:** 2021-12-22

**Authors:** Nicholas Sawers, Nigel Bolster, Andrew Bastawrous

**Affiliations:** ^1^The International Centre for Eye Health (ICEH), London School of Hygiene and Tropical Medicine, London, United Kingdom; ^2^Peek Vision, London, United Kingdom

**Keywords:** eye health, artificial intelligence, machine learning, public health research, m-Health

## Abstract

Achieving The United Nations sustainable developments goals by 2030 will be a challenge. Researchers around the world are working toward this aim across the breadth of healthcare. Technology, and more especially artificial intelligence, has the ability to propel us forwards and support these goals but requires careful application. Artificial intelligence shows promise within healthcare and there has been fast development in ophthalmology, cardiology, diabetes, and oncology. Healthcare is starting to learn from commercial industry leaders who utilize fast and continuous testing algorithms to gain efficiency and find the optimum solutions. This article provides examples of how commercial industry is benefitting from utilizing AI and improving service delivery. The article then provides a specific example in eye health on how machine learning algorithms can be purposed to drive service delivery in a resource-limited setting by utilizing the novel study designs in response adaptive randomization. We then aim to provide six key considerations for researchers who wish to begin working with AI technology which include collaboration, adopting a fast-fail culture and developing a capacity in ethics and data science.

## Introduction

Eye health is a development issue. Reports in 2020 estimate that Worldwide, 596 million people had a distance visual impairment problem, of whom 43 million were blind. A further 510 million had uncorrected near vision impairment (1). Improving eye health unequivocally leads to improvement in quality of life, reducing inequality, education and income (2). Eye health is key to ensuring good health, mental health and well-being (3) and an increasing body of evidence shows how central eye health is to advancing the United Nations (UN) sustainable development goals (SDGs). Eye health contributes to multiple SDGs as the benefits are not only limited to improving sight, but also reducing disability and morbidity. In addition to contributing to health (SDG3 “Good health and well-being”), eye health reduces poverty and subsequently reduces hunger (SDG2 “Zero hunger”) (4). Furthermore, a study of the economics of eye health show the impact of vision loss on productivity and employment (SGD8 “Decent work and economic growth”) (5). The first UN Resolution on Vision was adopted by all 193 member states in July 2021, further confirming its importance globally (6).

Universal Health Coverage (UHC) is one of the overarching targets of the 2030 agenda for sustainable development. UHC states that anyone who needs health care can access quality health services without risk of financial harm. This means effective, accessible, high quality and integrated healthcare which is often lacking in many settings (7). Effective coverage is often cited as a metric for UHC monitoring but many health systems are unable to measure it. Effective coverage aims to add impact through measuring need, use and quality to better capture the benefits of an intervention (8). Eye care is integral to achieving UHC. There has been a global commitment to measuring UHC in eye health with two metrics adopted at the World Health Assembly (9) [effective cataract surgery coverage (eCSC) and effective refractive error coverage (eREC)]. These two metrics will act as a proxy for the status of eye care services and contribution toward UHC across multiple countries. Achieving these goals and measuring these metrics is challenging (10) and requires further work on implementation strategies (11).

To tackle this, improvement science, the study of how to implement positive change, aims to introduce systematic methodologies from industry corporations such as Toyota (12). The field has been developed from commercial industry to be widely used in healthcare and aims to provide a framework to deliver change and achieve sustained improvement (13). The emergence of Healthcare 4.0, artificial intelligence (AI), personalized medicines, and telemedicine with cyber sensors (14), is providing the foundation to apply vast amounts of data to management decisions and improvement science (15). We have witnessed an exponential growth in technology in the last decade and Covid-19 has fastened the requirement for health systems to scale up, and expand the applications of digital innovations (16) AI is at the forefront and will continue to rise and come into every facet of our lives. The largest and most successful companies in the world such as Google and Amazon utilize AI, learning constantly and testing inherently in a culture of accepted failure (17). Healthcare is again developing the use of implementing change through AI in commercial industry and applying the technology in a variety of settings. Medicine is behind, but there are shoots in cancer care (18), cardiology (19) and diabetes (20) that are pioneering the future of this space. Additionally, AI has recently been used to help countries respond to the Covid-19 pandemic (21).

For clarity, AI is a vast field that aims to develop computers with the capabilities of humans. Machine Learning (ML), a subset of AI, is one route to achieve it. ML studies algorithms that allow computers to automatically improve through continuing experience (22). Computer programs can learn and adapt by training algorithms to provide insights, classifications and predictions from data sets.

Within eye health in particular there has been variety of AI based interventions including screening retinal photographs for diabetic retinopathy, cataract surgery decision making and the use of AI within telemedicine to help reach low resource settings (2). Within eye health, the main causes of morbidity have clear screening protocols, diagnostic tools and treatments that mean the key hurdles are in service delivery; ensuring the right people get the right care at the right time (2). We must be able to apply new technology and improvement methodologies to increase access, quality and coverage across health systems. However, we will need flexible and adaptable methods to address a wide range of local system challenges. Although there has been an explosion of data, methodologies and large collaboratives to solve problems, this doesn't necessarily lead to better outcomes (23, 24). The introduction of digital health technologies will require careful application to ensure both equitable delivery and progress toward achieving the SDGs (2).

This article begins by highlighting several commercial industry examples of AI use in developing and testing solutions. We then aim to look in detail at how AI can be used in testing strategies and provide a case study within eye health where ML could be employed, and detail a set of considerations for researchers that are looking to expand into a novel way of optimizing their study designs. These considerations focus on the initial stages of working with ML and beginning to plan to use study designs or a testing strategy that utilizes the technology. Furthermore, there are many detailed explanations of the algorithmic considerations and study designs using adaptive design that are out of the scope for this article, but are a valuable read. Papers by Kaiblel and Biemann (25) and Ajmera et al. (26) provide a foundation for the use of adaptive study designs, and Villar et al. (27) and Williamson and Villar (28) provide further detail on adaptive algorithms.

## AI Use in Commercial Industry

Incremental gains in service delivery has been the focus of the aviation and automobile industries for decades (29) and has resulted in gradual improvement over a long periods of time. This philosophy has been adopted in recent years by the healthcare community with continuous improvement and implementation research (30, 31). However, industry-led improvement science has evolved again and large internet-based companies such as Amazon and Google utilize AI and more specifically machine learning (ML) for improving their business models and developing new solutions. For example, over the past decade, ML has given us self-driving cars (32), practical speech recognition (33), effective web search (34), and a vastly improved understanding of the human genome (35). Now we are seeing technology drive improvements in healthcare (36).

Some of the largest companies in the world: Amazon, Spotify and Google amongst many, use ML to apply a relentless foray into testing at large scale with a willingness to improve on a “no shame in failure” basis (17). Large companies are harnessing big data to speed up the journey from information and insights to decisions and actions (37). In a shareholder letter in 2016, Jeff Bezos told Amazon employees that his company was the best place in the world to “fail,” allowing their systems to learn continually (38). Arguably the opposite culture is seen in medical research where the focus is on providing positive results and evidence of failure is often not published. These large companies such as Netflix utilize ML to learn continuously from their customer's habits, building personalized homepages wherein the more you use the platform, the more accurate the system reflects your preferences. Many of Amazon's sales are due to their recommendation widget which is a ML algorithm tested on millions of customers, trialing new ideas continually. The ability to continuously test multiple solutions at the same time has fast tracked improvements.

## Testing With Machine Learning

The randomized control trial (RCT) has been the gold standard in medicine since the late twenty-first century (39). RCTs are a robust form of evidence-based testing and are widely acknowledged to generate the highest level of evidence. RCTs require careful consideration and rigorous planning and coordination (40). The intricate trial minimizes bias and the influence of confounding factors. However, all scientific methods have weaknesses which have been debated at length in the literature, especially RCTs and measuring improvement (41). The Hippocratic maxim “do no harm,” is one of the cornerstones of modern medicine. Within the realm of research, this means that no harm should be inflicted upon anyone, regardless of the benefits. However, on a population level, this means a highly constrained testing environment. What harm are we causing by not learning and rapidly adapting? All learning requires an element of risk exposure, how we manage this risk whilst incorporating AI will be an important hurdle for evidence-based testing in healthcare.

RCTs are by their nature designed to succeed, in part because of the high cost and incentive to publish results. This article by no means wishes to debunk RCTs, but instead argues for a real-world adjuvant testing strategy which can overcome some of the hurdles that traditional RCTs come across in sustained and complex service delivery. The type of challenges particularly affects the delivery of healthcare services globally with the most affected being in LMICs. Firstly, the intensive commitment of resources and time is a restraint in many study designs. And secondly, the lack of a real world setting to test interventions and hypotheses.

Response adaptive randomization (RAR) is an alternative study design to measure continuous improvement (42). RAR is an industry standard with companies such as Spotify making use of their large data sets to continuously test interventions in the form of A/B testing. These companies have gone a step further and now use reinforced learning (RL), a subset of ML, to test interventions (43). At multiple stages, data analysis is conducted and adaptations to the testing strategy take place based on continually updated information to maximize the probability of success of an intervention being selected. ML RAR trials are increasing in number every year, but the sample sizes have been small and the data collection not fully integrated. As the trial proceeds, the incoming data informs the randomization of the following allocations of interventions. The trial algorithm selects the optimal intervention using data-dependent sampling. RAR allows for faster testing of hypotheses and a smaller sample size. However, it is worth noting that the study design and analysis of results has to be carefully planned. Studies with RAR that succeed with patient-benefits with unbiased and statistically rigorous comparison of the different treatments are challenging (44).

RCTs perform excellently with competing treatment options for evidence-based patient care. However, health systems are inherently complex and therefore involve a plethora of factors that evolve as time progresses (45). Some trials using a RCT design require extremely large sample sizes which may be difficult for recruitment and the designs are often different from the real world complexity in a health system. Measuring how a system improves, key to service delivery, is more suited to health systems. ML can provide an option to measure and test improvements incrementally over time.

## Machine Learning Testing in Health Systems

Each health system is different and inherently complex (46). The interplay between patients, healthcare workers and support staff in a variety of different environments ensure outcomes are unpredictable. This is especially true for large, complex systems that deal with a variety of health conditions, locations and demographics that require a deep understanding of the underlying frameworks to develop effective research approaches (45). It is well established that we now have the diagnostic tools and effective treatments for many non-communicable diseases in the world. For example, in eye health, although 90% of those affected by vision impairment live in LMIC countries, 90% of those are affected by treatable or have preventable causes with cost effective interventions (2). What is required in many low and middle-income countries (LMICs) is effective, high quality service delivery (47). Ensuring the right treatment to the right people at the right time is challenging, especially when there are fundamental aspects of human resource shortages, challenging environments and places with poor access to medical care due to either transport or economic hurdles (48). How to effectively implement service delivery is key and an area where machine learning testing can be used.

Health systems such as a hospital with screening, diagnostics and management functions spread across various geographical sites with different actors more similar to other service delivery systems such as Amazon than a single drug which is inherently more suited to RCT style testing. As we have seen in the last decade, healthcare has applied industry standard continuous improvement methodology to optimize performance (13). There is currently little information on implementing artificial intelligence in eye health delivery and even less on whether it could improve care or outcomes. Can we apply industry-standard testing designs such as ML algorithms to improve service delivery of already proven interventions in our health systems as well as optimization of those systems?

## An Example in Eye Health

With the influx of data from smartphones, we are now able to monitor and evaluate data in real time. For example, Peek Solutions use mobile phone applications and software for eye health screening which has been specifically designed for eye health programmes in low-resource setting (49). Integrated, automated and personalized SMS texting is effectively used to improve the adherence of school children coming to appointments for eye exams after screening. The trial that showed this effectiveness took 24 months to complete and a further 18 months to disseminate through publication. The transparency of data from the use of smartphones at screening, diagnostics and treatment in a patient's pathways gives a live real-time view of the health system with any potential blockers or areas for improvement. This data transparency allows programme managers to focus on improvement areas. However, how can we be sure that interventions to the system are clinically relevant?

If we take the example of SMS communication, peek solutions have shown in a cluster RCT in Kenya that SMS reminders increase the adherence of patients to eye examinations providing an improvement in service delivery (50). Following on from this work, we can apply similar testing regimes to industry leaders to find out the optimal solution to test interventions for SMS reminders. For example, attempting to find out what frequency, mode (voice/message), motivation style to use whilst the real world programme is running. The more testing performed, the better the system learns and the more accurate it becomes over time. If a particular intervention is working, then the algorithm favors this intervention over another. The DIAMANTE study protocol in diabetic research aims to tailor SMS interventions using ML methods to increase the effectiveness of the intervention (20). Over time, we could add intervention arms to the existing system and assess whether the system applies the new intervention at scale. One can utilize RAR, but the randomization is driven by evidence-based ML protocols.

The advantages of this approach are plentiful. The main advantages are time and resources. ML algorithms are faster at selecting the optimal intervention than an RCT design and democratizes the decision making to local programme leaders. Focusing on flexible solutions for local needs gives more responsibility for teams to make decisions for their areas. This in turn should lead to improvement in decision making with the additional knowledge of local factors which should increase efficiency and quality of service delivery to those who need it. Once an algorithm has been designed and tested, programme teams can select the interventions they wish to test and the system will learn the optimum outcome. However, this approach is not without challenges. There is a lot of preparation work in order to successfully implement this type of system but the benefits of running a testing system within a live running programme are game changing.

Large tech corporations often shape every problem as something that will inevitably have a technological solution. That's clearly not true (51). Many public health systems require a foundation to build highly effective, large scale testing centers. Furthermore, a testing system that evaluates the optimal solution has to be in a way that is also affordable, safe and ethical.

## Considerations for Machine Learning Research

As a research group, we have worked toward implementing a ML based testing strategy for a health system in a low-middle income country. In reflection, highlight six considerations that we believe would benefit other researchers beginning to work within the field of ML.

### Learn From Leaders in Industry—The “Fail Fast” Culture

Lead the culture change in science away from the restrictive definitions of “success,” and toward deeper learning whereby testing interventions and failure is part of the journey and should be revered as much as positive interventions. This change in mindset is a necessity to drive an environment of perpetual testing and align with ML protocols. Positive and negative results should be published and celebrated alike.

### Collaborate With ML Researchers

Although selecting an optimal algorithm for research in different clinical environments is feasible, the interpretation and judgement for implementing algorithms are very challenging. Developing the necessary deep understanding of statistics and knowledge in ML is a challenge and therefore, we recommend early collaboration with leading researchers in the field to make this transition.

### Integrate and Develop Health Data Science Courses

Healthcare and data science will be intrinsically linked for decades to come as technology drives change and performance gains. However, we need to ensure that researchers are closely integrated with graduates and courses so that we can learn from each other. It will be important to develop the next generation of health data science graduates with a focus on impact and service delivery whilst also developing the course in the direction that will be most advantageous for future research and implementation. Developing projects and dissertations that link data science courses and research teams can provide a good opportunity for integration and learning.

### Capacity Building in LMICs in Technology-Based Testing

There are plenty of examples of tech hubs in LMICs with a focus on driving improvement such as the Botswana Innovation Hub and Technology Innovation Centre in Nigeria which aim to develop and nurture businesses who create impact with technology innovations (52). This needs to be reflected within LMIC healthcare teams where desirable skills sets include experience with technology and ML. Building capacity for local teams to drive innovation will be an important step for a sustainable future in technology-led testing.

### Develop Ethics Knowledge and Regulatory Framework

The debate around ethics in RAR trials will continue. Many cite ethics as a reason to use RAR trials in their study design and the discussion will continue (42). Developing a strong understanding of the ethics of using ML will be imperative to successfully advocating this type of testing strategy. For example researchers will have to decide how much autonomy of decision making is ethically appropriate for algorithms to make and whether the use of stopping rules or interim analysis checks are required. Working closely with ethics teams to provide the regulatory framework in order to be ready for when the technology is ready to deliver will be important.

### Develop an Understanding of ML Principles Within the Research Community

The use of ML is exciting. The technology provides an opportunity to drive improvement across many domains. Advocacy will be an important factor in the journey so we recommend bringing ML to team research meetings, topics of interest, and research conversations to involve the wider community and normalize the field, especially around the people centered advantages of technology, not just technological advancement itself.

## Conclusions

The question to which we wish to conclude upon is “what will give public health the best chance of taking advantage of the exponential rise in the technology of the twenty-first century to better deliver healthcare and achieve the SDGs by 2030?”

Although the Covid-19 pandemic has caused a slowdown in the advancements on public health, we can take this opportunity to take stock and evaluate where the biggest gains will be made in the next 10 years. This article argues that the integration of ML alongside traditional study designs into public health can propel testing and sustained incremental gains championed by improvement science. We need to learn lessons from industry leaders and predict where the world is heading and what our future health system could encompass.

We highlight several key considerations to plan for the successful application of machine learning algorithms when applying AI to eye health programmes and testing strategies. However, this space is relatively new and researchers will need to evolve as the field matures. Healthcare organizations will be required to adapt to the technology advancements within AI whilst keeping abreast of the SDGs and ensuring impactful change.

Health systems built to utilize the advantages of ML through perpetual testing will be a challenge. In order to achieve this, we need to build capacity within existing public health systems by collaborating with existing ML researchers and developing specific ML testing knowledge within research teams. With the increasing use of ML in RAR study designs and data science more generally, this presents an opportunity for public health, and more specifically public eye health as technology is being used in population based screening and diagnostics. Achieving the SDGs by 2030 will be challenging and in order to check progress there will need to be continued reviews and reflections on progress so that researchers and implementers can refine and evaluate their work. Ensuring we work toward that focus of achieving the SDGs is a start and keeping track of metrics to evaluate effective coverage and UHC within objectives for eye health programmes will support that goal. We additionally need to embrace and lead developments with AI and ML in both theory and practice to work toward optimal service delivery and achieving multiple SDGs through universal health coverage.

## Data Availability Statement

The original contributions presented in the study are included in the article/supplementary material, further inquiries can be directed to the corresponding author.

## Author Contributions

NS wrote the manuscript. AB and NS contributed to the content of the manuscript. All authors reviewed the final manuscript.

## Funding

This work was supported by the National Institute for Health Research (NIHR) [using the UK's Official Development Assistance (ODA) Funding] and Welcome (grant 215633/Z/19/Z) under the NIHR-Wellcome Partnership for Global Health Research.

## Author Disclaimer

The views expressed are those of the authors and not necessarily those of Welcome, the NIHR or the Department of Health and Social Care.

## Conflict of Interest

NB and AB were employed by company Peek Vision, London, United Kingdom. The remaining author declares that the research was conducted in the absence of any commercial or financial relationships that could be construed as a potential conflict of interest.

## Publisher's Note

All claims expressed in this article are solely those of the authors and do not necessarily represent those of their affiliated organizations, or those of the publisher, the editors and the reviewers. Any product that may be evaluated in this article, or claim that may be made by its manufacturer, is not guaranteed or endorsed by the publisher.
